# Clinical Features of Fatalities in Patients With COVID-19

**DOI:** 10.1017/dmp.2020.235

**Published:** 2020-07-14

**Authors:** Ya-Jun Sun, Yi-Jin Feng, Jing Chen, Bo Li, Zhong-Cheng Luo, Pei-Xi Wang

**Affiliations:** Institute of Chronic Disease Risks Assessment, Henan University, Jinming Campus, Kaifeng, China; Department of Obstetrics and Gynecology, Lunenfeld-Tanenbaum Research Institute, Prosserman Centre for Population Health Research, Mount Sinai Hospital, Institute of Health Policy, Management and Evaluation, Dalla Lana School of Public Health, Faculty of Medicine, University of Toronto, Toronto, Canada

**Keywords:** clinical characteristics, COVID-19, fatality

## Abstract

**Objectives:**

The novel coronavirus disease 2019 (COVID-19) pandemic has spread to over 213 countries and territories. We sought to describe the clinical features of fatalities in patients with severe COVID-19.

**Methods:**

We conducted an Internet-based retrospective cohort study through retrieving the clinical information of 100 COVID-19 deaths from nonduplicating incidental reports in Chinese provincial and other governmental websites between January 23 and March 10, 2020.

**Results:**

Approximately 6 of 10 COVID-19 deaths were males (64.0%). The average age was 70.7 ± 13.5 y, and 84% of patients were elderly (over age 60 y). The mean duration from admission to diagnosis was 2.2 ± 3.8 d (median: 1 d). The mean duration from diagnosis to death was 9.9 ± 7.0 d (median: 9 d). Approximately 3 of 4 cases (76.0%) were complicated by 1 or more chronic diseases, including hypertension (41.0%), diabetes (29.0%) and coronary heart disease (27.0%), respiratory disorders (23.0%), and cerebrovascular disease (12.0%). Fever (46.0%), cough (33.0%), and shortness of breath (9.0%) were the most common first symptoms. Multiple organ failure (67.9%), circulatory failure (20.2%), and respiratory failure (11.9%) are the top 3 direct causes of death.

**Conclusions:**

COVID-19 deaths are mainly elderly and patients with chronic diseases especially cardiovascular disorders and diabetes. Multiple organ failure is the most common direct cause of death.

In December 2019, several cases of pneumonia of unknown cause were reported in Wuhan, China that were later recognized as a novel coronavirus infection, named coronavirus disease 2019 (COVID-19) by the World Health Organization (WHO).^1^ COVID-19 has been included in the laws of the People’s Republic of China in the prevention and treatment of infectious diseases as a class B infectious disease. All provinces and cities in China have taken first-level public health emergency responses to contain the transmission of the disease and protect vulnerable populations. The epidemic has spread across China as well as into 213 countries and territories.^2^ The COVID-19’s socioeconomic impacts have already far exceeded those of severe acute respiratory syndrome (SARS) and the Middle East respiratory syndrome (MERS), and the pandemic has become a worldwide major public health concern. As of June 21, 2020, the global number of confirmed cases of COVID-19 exceeded 9 million, leading to over 470 thousand of fatalities.^2^ We attempted to describe the clinical characteristics of fatalities in patients with COVID-19, which may inform the clinical management of patients with severe COVID-19.

## METHODS

This was an Internet-based data intelligence study. We constituted a cohort of COVID-19 deaths through retrieving the clinical information on COVID-19 fatalities from nonduplicating incidental reports in Chinese provincial and metropolitan city Health Commission and other governmental official websites between January 23 and March 10, 2020. The reported clinical characteristics included the patient’s age, sex, initial onset symptoms, pre-existing chronic diseases, direct cause of death, date of admission, date of diagnosis, and date of death. The study cohort included 100 cases of COVID-19 fatalities. The study was approved by the research ethics committee of Henan University. Informed consent was waived, because the study was based on publicly available anonymized incidental fatality reports.

### Statistical Analysis

SPSS (version 22.0) software was used for statistical analysis. Mean ± standard deviations (SD) and median (inter-quartile range) were presented for continuous variables, while frequency and percentage were presented for categorical variables.

### Patient and Public Involvement

Patients were not involved in the study, which is based on anonymized incidental COVID-19 fatality reports from governmental websites.

## RESULTS

Approximately 6 of 10 COVID-19 fatalities (64.0%) were males (Table 1). The average age was 70.7 ± 13.5 y (median: 72.5 y), and approximately 8 of 10 patients (84.0%) were over 60 y of age. The mean duration from admission to diagnosis was 2.2 ± 2.8 d (median: 1). The average duration from the diagnosis to death was 9.9 ± 6.5 d (median: 9 d).

**TABLE 1:** Clinical Characteristics of COVID-19 Fatalities (*n* = 100)

	Mean ± SD	*N* (%)	Median (IQR)
Age (years)	70.7 ± 13.5		72.5 (65.0,80.8)
≥60		84 (84.0)	
Sex, male		64 (64.0)	
Days from admission to diagnosis	2.2 ± 3.8		1.0 (0.0, 2.8)
Days from diagnosis to death^a^	9.9 ± 7.0		9.0 (4.0,13.0)
Chronic illness, any		76 (76.0%)	
Hypertension		41 (41.0)	
Diabetes		29 (29.0)	
Cardiovascular disease		27 (27.0)	
Respiratory disorders		23 (23.0)	
Cerebrovascular disease		12 (12.0)	
2 or more chronic illnesses		48 (48.0)	
Signs and symptoms at onset			
Fever		46 (46.0)	
Cough		33 (33.0)	
Shortness of breath		9 (9.0)	
Fatigue		8 (8.0)	
Sputum		7 (7.0)	
Dyspnea		7 (7.0)	
Direct cause of death^b^			
Multiple organ failure		57 (67.9)	
Circulatory failure		17 (20.2)	
Respiratory failure		10 (11.9)	

a A total of 12 cases were missing data on the time from diagnosis to death.

b A total of 16 cases were missing data on direct cause of death.

Approximately 3 of 4 fatal COVID-19 cases (76.0%) had 1 or more pre-existing chronic illnesses. The prevalence rates were 41.0% for hypertension, 29% for diabetes, 27.0% for coronary heart disease, 23% for respiratory disorders, 12% for cerebrovascular disease, 3% for cancers, 5% for abnormal renal function, and 2.0% for Parkinson’s disease. Approximately half of patients (48%) had 2 or more chronic diseases.

Fever (46.0%), cough (33.0%), shortness of breath (9.0%), fatigue and weakness (8.0%), sputum (7.0%), and dyspnea (7.0%) were the common symptoms at onset, whereas palpitations and diarrhea were less frequent. Among the 100 COVID-19 fatalities, 16 cases were missing data on direct cause of death. Of the 84 COVID-19 cases with known direct cause of death, the top 3 common direct causes of death were multiple organ failure (67.9%), circulatory failure (20.2%), and respiratory failure (11.9%), and were similar (*P* = 0.50) for males and females: multiple organ failure (64.8% vs 73.3%, respectively), circulatory failure (24.1% vs 13.4%, respectively), and respiratory failure (11.1% vs 13.3%, respectively).

## DISCUSSION

In this Internet-based data intelligence study, we observed that the majority of COVID-19 deaths were elderly (approximately 8 of 10) and males (6 of 10), and most fatalities (3 of 4) occurred in patients with chronic illnesses. The findings were consistent with a recent report in a hospital-based study,^3^ and with the WHO report on COVID-19 in China,^4^ and demonstrate the usefulness of an Internet-based data intelligence study. Previous studies have not clarified the direct causes of death. Our data indicate that the most common direct cause of death is multiple organ failure (approximately 2 of 3). The initial onset symptoms are not so much saliently worrisome, but the median duration from diagnosis to death was only 9 d, indicating that the disease can worsen rapidly, costing life.

The function of innate immunity and neutrophil function may degrade with aging, exposing the elderly to the more deleterious impact of the new coronavirus infection. Similar to SARS and MERS, COVID-19 presents a clear male sex bias.^5^ Compared with males, the immune response in females may be more vigorous with higher antibody levels following exposure to an infectious agent;^6^ thus, females may be less vulnerable to the deleterious consequence of COVID-19 infection. It has been speculated that women’s lower susceptibility to viral infections may be related to genetic factors associated with the X chromosome and sex hormones. Another possible explanation for the higher incidence and more male COVID-19 fatalities may be due to that males are likely to spend more time outdoors, increasing the chances of exposure to the virus.

Consistent with previous reports, most (approximately 3 of 4) fatalities occurred in patients with chronic illnesses.^3,7^ The top 4 were hypertension (41.0%), diabetes (29.0%), coronary heart disease (27.0%), and respiratory disorders (23.0%). Previous studies indicate that COVID-19 shares the same receptor with SARS-COV, and the angiotensin-converting enzyme-2 (ACE2) sensitive cell surface receptors mediate the entry of the virus into the target cells.^8^ ACE2, the functional receptor of SARS-COV, is expressed in the islet, through which the virus may invade and destroy the pancreatic islet cells, thus may aggravate diabetes and accelerate the disease progression. The immune system plays a crucial role when the body is confronted with viruses or bacteria. For patients with diabetes, especially those with poor blood glucose control, long-term exposure to hyperglycemia may lead to decreased immune function. Other chronic illnesses may also compromise the patient’s immune defense system leading to severe consequences.

Multiple organ failure, respiratory failure, and circulatory failure were the main direct causes of deaths. Similar to MERS-CoV,^9^ multiple organ failure appears to be a common direct cause of death in COVID-19 fatalities. The COVID-19 infection may lead to increased blood capillary permeability of the lungs,^10^ aggravating inflammation and apoptosis, with lung injuries leading to respiratory distress syndrome. The virus may set off an immune inflammatory response storm, causing tissue damages in multiple organs leading to multiple organ failure.

This study has some limitations. First, we did not have the laboratory data in this Internet reports-based study. The reported clinical characteristics are relatively limited in Internet reports. It is unclear whether there is a selection bias in Internet reports of COVID-19 fatalities compared with those fatalities in the general population. However, our data on age and sex distributions of COVID-19 deaths are consistent with the recent report on 113 deaths in a single large hospital-based study in Wuhan, China.^3^

In conclusion, COVID-19 deaths are mainly elderly and patients with chronic diseases, especially cardiovascular disorders and diabetes. Multiple organ failure is the most common direct cause of death. Our findings may inform clinical health-care professionals in better management of severe COVID-19 patients in fighting the emerging pandemic.

## Data Availability

The study data are available from the corresponding author upon reasonable request.

## References

[1] Huang C , Wang Y , Li X , et al. Clinical features of patients infected with 2019 novel coronavirus in Wuhan, China. Lancet. 2020;395(10223):497-506. doi: 10.1016/S0140-6736(20)30183-5 31986264 PMC7159299

[2] Worldometer. COVID-19 coronavirus pandemic. https://www.worldometers.info/coronavirus/. Accessed June 21, 2020.

[3] Chen T , Wu D , Chen H , et al. Clinical characteristics of 113 deceased patients with coronavirus disease 2019: retrospective study. BMJ. 2020;368:m1091. doi: 10.1136/bmj.m1091 32217556 PMC7190011

[4] WHO. Report of the WHO-China Joint Mission on Coronavirus Disease 2019 (COVID-19). https://www.who.int/docs/default-source/coronaviruse/who-china-joint-mission-on-covid-19-final-report.pdf. Accessed July 11, 2020.

[5] Channappanavar R , Fett C , Mack M , et al. Sex-based differences in susceptibility to severe acute respiratory syndrome coronavirus infection. J Immunol. 2017;198(10):4046-4053. doi: 10.4049/jimmunol.1601896 28373583 PMC5450662

[6] Moxley G , Posthuma D , Carlson P , et al. Sexual dimorphism in innate immunity. Arthritis Rheum. 2002;46(1):250-258. doi: 10.1002/1529-0131(200201)46:1<250:AID-ART10064>3.0.CO;2-T 11817599

[7] Onder G , Rezza G , Brusaferro S. Case-fatality rate and characteristics of patients dying in relation to COVID-19 in Italy. JAMA. 2020. doi: 10.1001/jama.2020.4683 32203977

[8] Lu R , Zhao X , Li J , et al. Genomic characterisation and epidemiology of 2019 novel coronavirus: implications for virus origins and receptor binding. Lancet. 2020;395(10224):565-574. doi: 10.1016/S0140-6736(20)30251-8 32007145 PMC7159086

[9] Zumla A , Hui DS , Perlman S. Middle East respiratory syndrome. Lancet. 2015;386(9997):995-1007. doi: 10.1016/S0140-6736(15)60454-8 26049252 PMC4721578

[10] Ackermann M , Verleden SE , Kuehnel M , et al. Pulmonary vascular endothelialitis, thrombosis, and angiogenesis in Covid-19. N Engl J Med. 2020;383(2):120-128. doi: 10.1056/NEJMoa2015432 32437596 PMC7412750

